# Correlation Between Central Corneal Thickness and Radial Peripapillary Capillary Density, in Patients With Ocular Hypertension

**DOI:** 10.7759/cureus.17138

**Published:** 2021-08-12

**Authors:** Elpida Kollia, Eleni Patsea, Ilias Georgalas, Dimitrios Brouzas, Dimitrios Papaconstantinou

**Affiliations:** 1 Ophthalmology, National and Kapodistrian University of Athens School of Medicine, Athens, GRC; 2 Ophthalmology/Glaucoma, Ophthalmiatreion Athinon, Athens, GRC; 3 Ophthalmology, "G. Gennimatas" Hospital, National and Kapodistrian University of Athens School of Medicine, Athens, GRC

**Keywords:** central corneal thickness, radial peripapillary capillary density, optical coherence tomography angiography, ocular hypertension, risk factors

## Abstract

Purpose

To investigate any possible relationship between the central corneal thickness and the radial peripapillary capillary density detected by optical coherence tomography (OCT) angiography in eyes with ocular hypertension.

Materials and methods

In this observational study, 135 eyes were examined. OCT angiography of the optic disc (4.5 mm) and ultrasound corneal pachymetry were performed in all cases. Age, medical treatment for ocular hypertension, sex, and retinal nerve fiber layer thickness were evaluated. The main indices of blood flow were also examined. Spearman correlation coefficients were used to explore the association between two continuous variables.

Results

A directly proportional significance regarding the correlation between central corneal thickness and radial peripapillary network was indicated in eyes with ocular hypertension (p = .036).

Conclusions

Central corneal thickness and radial peripapillary capillary density constitute two essential screening parameters for patients with ocular hypertension.

## Introduction

Ocular hypertension (OH) is defined as a condition in which the intraocular pressure (IOP) is greater than 21 mmHg in one or both eyes, with no evident glaucomatous defect. Its prevalence in people older than 40 years is estimated to range from 3% to 10% [1-4]. High IOP is a risk factor for conversion to primary open-angle glaucoma. Increased intraocular pressure, advanced age, myopia, thin cornea, and dark skin color are the main risk factor [5-11]. The mean central corneal thickness (CCT) is higher in eyes with ocular hypertension than in normal or glaucomatous eyes. As reviewed in the Ocular Hypertension Treatment Study (OHTS), CCT may affect the accuracy of applanation tonometry in the screening and clinical treatment of patients with glaucoma and ocular hypertension. Hence, it has been studied in terms of its direct interrelationship with ocular pressure [2,8-11]. The recently released technology of optical coherence tomography angiography (OCT-A) constitutes a novel, non-invasive imaging method that detects blood flow in the retina and optic nerve head. It uses variations in the intensity and/or phase properties of the OCT signals that result from the movement of blood over multiple B-scans to generate a high-resolution map of the microcirculation. Thus, OCT-A provides a quick and meticulous evaluation of retinal vascular structures. Specifically, the radial peripapillary capillary plexus was assessed as far as the optic nerve head [12-18]. The principal aim of our study was to substantiate any correlation between CCT and radial peripapillary capillary density (RPC) in patients with ocular hypertension in terms of early and thorough clinical assessment of this entity.

## Materials and methods

In this study, 135 eyes with ocular hypertension were examined. Data were collected prospectively during one year. All subjects were adults over 18 years of age. Patients (eyes) with the following conditions were excluded from the study: anti-glaucoma treatment with more than one pharmaceutical agent, any type of treatment for ocular diseases, dense cataracts, conditions consistent with abnormal macular findings, signs of uveitis, previous complicated cataract surgery, previous vitreoretinal interventions, previous refractive operations, and selective laser trabeculoplasty procedures.

The same protocol was applied to all the cases. First, all patients were carefully examined clinically. They underwent medical history recording, visual acuity evaluation, slit-lamp examination, tonometry, and fundus examination. Afterwards, OCT-A of the optic disc (4.5 mm program) and ultrasound corneal pachymetry were performed in all eyes. AngioVue HD (Optovue, Fremont, CA) software was used for all angiography measurements.

With regards to the applied statistical analysis, normally distributed variables were expressed as means (standard deviation), while variables with skewed distribution were expressed as median (interquartile range). Qualitative variables were expressed as absolute and relative frequencies. Spearman correlation coefficients were used to explore the association between two continuous variables. All reported p-values were two-tailed. Statistical significance was set at p < 0.05, and analyses were conducted using SPSS statistical software version 22.0 (IBM Corp., Armonk, NY).

All methods were carried out in accordance with the relevant existing guidelines and medical regulations. All experimental protocols involved in the study included the actualization of clinical and screening examinations. Ethical approval was obtained from the General Assembly of Medicine School of National and Kapodistrian University of Athens, after evaluation by the Scientific Committee for Research Ethics of Athens Medicine School. Written informed consent was obtained from all participants.

## Results

**Figure 1:**
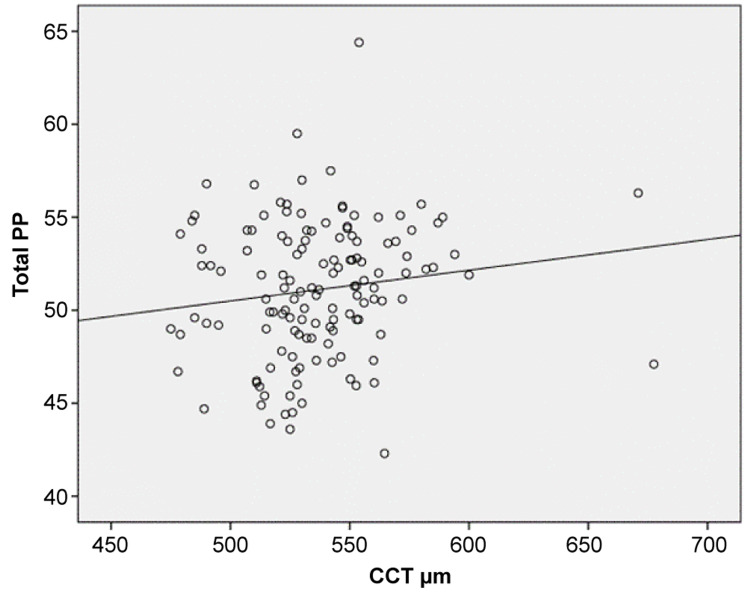
Correlation between total peripapillary capillary density (PP) and central corneal thickness (CCT) Spearman correlation coefficient (r) and p-value.

The sample consisted of 135 patients with a mean age of 62.1 years +/-13.1 SD (Table 1). Most patients (61.5%) were women. In addition, 47.4% of the patients were under medical treatment with one anti-glaucoma agent. The median C/D ratio was 0.40 (IQR: 0.30-0.50). The means and median values of the Retinal Nerve Fiber Layer (RNFL), CCT, and RPC measurements are presented in Table 2. Total peripapillary, superior-hemi, and inferior-hemi values of RPC were significantly positively associated with total peripapillary, superior-hemi, and inferior- hemi values of RNFL, indicating that higher RPC density values were significantly associated with higher RNFL values (Table 3). Inside disc values of RPC density were not significantly associated with RNFL values. Higher total peripapillary, superior-hemi, and inferior-hemi RNFL values were significantly associated with higher CCT values (p = .004; p = .009, and p = .030, respectively) (Table 4). The C/D ratio was only significantly and positively associated with CCT (p = .036). Furthermore, higher total peripapillary values were significantly associated with higher CCT values (p = .050) (Figure 1). Inferior RPC values tended to be positively associated with CCT values (p = .055).

**Table 1:** Patients’ characteristics SD: Standard deviation; IQR: Interquartile range; C/D ratio: cup-to-disc ratio.

	N (%)
Age (years), mean (SD)	62.1 (13.1)
Sex	
Women	83 (61.5)
Men	52 (38.5)
Under medical treatment	64 (47.4)
C/D Ratio, median (IQR)	0.40 (0.30 – 0.50)

**Table 2:** Description of RNFL, CCT and RPC measurements RNFL: Retinal nerve fiber layer; CCT: Central corneal thickness; RPC: Radial peripapillary capillary density; PP: Peripapillary; SD: Standard deviation; IQR: Interquartile range.

	Mean (SD)	Median (IQR)
Total RNFL	106.2 (9.9)	106 (99 - 111)
Superior	105.8 (10.9)	105 (97 - 114)
Inferior	106.7 (10.9)	105 (99 - 114)
CCT µm	536.7 (31.1)	534 (521.5 - 552.9)
Total PP	51.1 (3.6)	51.2 (48.7 - 53.9)
Superior	51.3 (4.2)	51.5 (48.3 - 54.0)
Inferior	50.7 (4.6)	50.9 (48.3 - 53.9)
Inside disc	50.5 (5.9)	51.1 (46.8 - 54.5)

**Table 3:** Spearman correlation coefficients between RNFL and RPC measurements *p < .05; **p < .01; ***p < .001 RNFL: Retinal nerve fiber layer; RPC: Radial peripapillary capillary density; PP: Peripapillary.

	Total RNFL	Superior	Inferior
Total PP	.41^***^	.40^***^	.30^***^
Superior	.38^***^	.40^***^	.26^***^
Inferior	.38^***^	.34^***^	.32^***^
Inside disc	-.01	.04	-.12

**Table 4:** Spearman correlation coefficients of RNFL and RPC measurements with CCT and C/D ratio *p < .05; **p < .01; ***p < .001 RNFL: Retinal nerve fiber layer; RPC: Radial peripapillary capillary density; CCT: Central corneal thickness; PP: Peripapillary; C/D ratio: cup-to-disc ratio.

	CCT - µm	C/D Ratio
Total PP	.17^*^	-.02
Superior	.13	.00
Inferior	.17	-.05
Inside disc	.13	-.06
Total RNFL	24^**^	.00
Superior	22^**^	-.08
Inferior	19^*^	.06
C/D Ratio	.18^*^	

## Discussion

OH has been an essential field of study. In the United States, its prevalence in non-Hispanic Whites over 40 years accounts for 4.5 percent, and increases up to 7.7 percent in 75- to 79-year-olds. In Latinos, the prevalence across ages is similar [18].

Risk factors and conditions related to OH have been meticulously studied. The Ocular Hypertension Treatment Study (OHTS), a multicenter randomized trial, remains the principal guiding light for all scientific approaches towards new immersive data. Its purpose was to evaluate the safety and efficacy of topical ocular hypotensive medical agents in delaying or preventing the onset of primary open-angle glaucoma (POAG) in individuals with elevated intraocular pressure (IOP) and no detectable glaucomatous damage.

The latest update of the OHTS indicates the baseline factors that predict the evolution of primary open-angle glaucoma. These include older age, race (African American), sex (male), larger vertical cup-disc ratio, larger horizontal cup-disc ratio, increased intraocular pressure, higher Humphrey visual field pattern standard deviation, heart disease, and thinner central cornea (CCT) [1-9, 1, 18-20].

Additionally, the RNFL assessment is widely used to weigh the amount of structural loss in OH and glaucoma, since the retinal thickness measurements present significant thinner change in patients suspected for glaucomatous conditions [18-19].

However, ocular blood flow changes have been considered to play a key role in the onset and development of different retinovascular and optic nerve diseases. Thus, not only RNFL thickness but also the density of RPCs network may also be associated with optic nerve head parameters and possible glaucomatous damage [21]. Furthermore, the ‘ISNT rule’, referring to the normal optic disc rim width (inferior ≥ superior ≥ nasal ≥ temporal) has also been used as a tool in screening for and diagnosing such entities [22].

In our study, after the evaluation of all parameters, significant interconnection between two essential parameters for the diagnostic algorithm of ocular hypertension parameters was encountered. There is an important correlation between CCT and RPC density. Moreover, after individually approaching the collected data, the low percentage of RPC density in eyes with OH was statistically significant. Specifically, a lower proportion of RPC density in the inferior hemi-quadrant of the optic nerve head in these eyes could potentially be associated with parallel or future structural changes. As a result, the possibility of conversion to primary open-angle glaucoma would be enhanced as the “ISNT” rim pattern (neural rim width: inferior ≥ superior ≥ nasal ≥ temporal) could be verified [15,11,18-22].

## Conclusions

In light of the above, the CCT and RPC density constitute two essential screening parameters for patients with ocular hypertension. After having taken all the aforementioned aspects into consideration, we can clearly conclude that the CCT and RPC density constitutes two associated parameters. We could assume that the newly introduced value of RPC density could be considered as an additional risk factor to those described in the OHTS. However, it would be interesting to examine this factor over a long-term period and with reference to scientific studies concerning primary open-angle glaucoma.
